# Venomous Snakes—A Request

**Published:** 1897-10

**Authors:** Alex. W. Stirling

**Affiliations:** Atlanta, Ga.


### VENOMOUS SNAKES—A REQUEST.

ATLANTA, GA., September 25, 1896.

*Atlanta Medical and Surgical Journal:*

It is probably well known that Dr. Calmette, of Lyons, and Dr. Fraser, Professor of Materia Medica in the University of Edinburgh, have for some time been endeavoring to produce a cure for poisoning by snake-bite by the production of an *alternated virus* for hypodermic use, and that in the laboratory at least their efforts have been crowned by considerable success. It may not, however, be so well known that Dr. Fraser has quite recently been experimenting with *bile* with the same object in view.* He first employed the bile from the snake itself, and found that a minute quantity injected under the skin of animals prevented the lethal effects of the venom. The active part of the bile he was able to separate by dissolving in water; it is insoluble in alcohol. He then proceeded to experiment with the bile of other animals, and discovered that that also possessed the desired power, even in minute quantities, though larger than those found sufficient when snake bile was employed. He ventured to deduce from these results that the same

* *Brit. Med. Jour.*, July 17, 1897.

constituents of the hepatic secretion would be found capable of destroying the toxins of diseases such as diphtheria, tetanus, etc.

Dr. Fraser has since given by subcutaneous injection* to a rabbit 0.15. c.cm. per kilo. of diphtheria toxin mixed with 0.05 gramme per kilo. of the dried bile of normal rabbits, and to a second rabbit 0.15 c.m. per kilo. of diphtheria toxin, but mixed with 0.025 gramme per kilo. of rabbit's bile. A month later both animals had gained in weight without even having a bad symptom except a slight rise of temperature. Three other rabbits received severally 0.15, 0.075 and 0.05 c.cm. per kilo. of the same toxin, and they were all dead in ten days.

When we consider that some 17,000 persons die annually in India alone from snake-bite, and that throughout the world the number is probably many times that figure, and when we farther consider what a deterrent the fear of snake-bite is, we get some idea of the importance of such work as that which is being carried on by Professor Fraser. (I say nothing farther here of its far more important relationship to diphtheria, tetanus, etc., as that lies without the scope of this communication.)

My principal object in writing now is to bring before the profession a request which I have received from Professor Fraser, that I would endeavor to procure for him the poison glands and gall-bladders of as many snakes as possible, for his ability to continue his investigations is limited by the difficulty of obtaining the necessary material. I shall here set down his directions for the removal of these parts of the snake's body, in order that any one who may have an opportunity of procuring recently killed snakes may, if he will be good enough to take the trouble, prepare the parts for transportation, and so aid in what may prove a very useful work. The venom glands are situated immediately beneath the skin of the upper jaw, below and posterior to the orbits, and can easily be exposed, their ducts tied, and the glands then dissected out. They can then be hung up in a warm (not hot), and if possible dry atmosphere, in which they soon become perfectly dry. The gall-bladder is particularly large in venomous snakes, but when dried with its

* *Brit. Med. Jour.*, September 4, 1897.

contents shrinks into a small bulk. It is situated in front, and a good way nearer the tail than the middle of the snake. The duct is long, and can easily be tied.

The dried venom-glands and gall-bladder of each snake should be tied together, and labeled, giving the name of the snake. If there is any doubt regarding the name it would be well to dry the head after removal of the venom-glands, and send it also.

Dr. Fraser has expressed his willingness to pay for these, and if the parts are sent to me I will make all arrangements necessary. I trust that what has been said will be sufficiently interesting to induce some members of the profession who live in districts in which snakes are to be found, to use their influence with such members of their community as may have a predilection for snake-hunting, in order that Professor Fraser's request may meet with an adequate response. ALEX. W. STIRLING, M.D.

